# Benefits of omalizumab on anxiety and depression in patients with severe asthma

**DOI:** 10.22088/cjim.9.3.228

**Published:** 2018

**Authors:** Fatih Uzer, Omer Ozbudak

**Affiliations:** 1 Department of Respiratory Medicine, Kastamonu State Hospital, Kastamonu, Turkey; 2 Department of Respiratory Medicine, Akdeniz University School of Medicine, Antalya, Turkey.

**Keywords:** Anxiety, Asthma, Depression, Omalizumab

## Abstract

**Background:**

Asthma is one of the most common chronic diseases and may cause psychiatric disorders affecting the patients’ quality of life. In our study, we evaluated the effect of omalizumab treatment on anxiety disorder and depression using Beck Depression Scale (BDS) and State Trait Anxiety Inventory (STAI).

**Methods:**

Anxiety level was determined with STAI, whereas depression level was evaluated by BDS. Patients were asked to mark the questionnaires to reflect their emotional state before treatment, and to reflect their emotions they are feeding in the current moment. All patients receiving omalizumab treatment were included in the study. Patients with known neuropsychiatric disorder were excluded from the study.

**Results:**

A total of 20 patients with mean age of 50.25 years were enrolled in the study. Gender distribution was: 5(25%) male patients and 15(75%) female patients. All patients with severe asthma received omalizumab treatment. The omalizumab treatment period was shown for mean 17.6 months (2-40 months). In anxiety scales, there was statistically significant difference compared with pretreatment and posttreatment periods. Depression (moderate to severe) was present in 12 patients before omalizumab treatment and 3 patients after omalizumab treatment.

**Conclusions:**

Uncontrolled asthma as a chronic disorder can cause depressive symptoms and worsen quality of life. We believe by controlling asthma, quality of life will improvein such patients. In appropriate indication, omalizumab can improve depression and anxiety in asthma patients.

Asthma is a major global health problem affecting over 300 million people of all ages worldwide and represents a significant socio-economic burden (1-3). It is stated in the literature that 5-10% of patients with asthma suffer from poorly-controlled disease despite corticosteroid treatment (4). It may cause psychiatric disorders via affecting the patients quality of life. Literature reports a significantly greater prevalence of mental disorders in people with asthma, with a particular emphasis on those with depression and/or anxiety (5-7). Bronchial hyperactivation, airway obstruction and related cough and shortness of breath can be seen in case of exposure to any agents being susceptible. All these symptoms can be overcome with complete or partial treatment. Living with the sense of shortness of breath may also affect the psychological status of the patient. In patients with worse asthma control, anxiety and depression are seen more commonly than normal population (3-9). The effects of the disease on the physical, social and psychological functions can be evaluated by quality of life questionnaire. In our study, we evaluated the effect of omalizumab treatment on anxiety disorder and depression by Beck Depression Scale (BDS) and State Trait Anxiety Inventory (STAI).

## Methods

This study was carried out between January-June 2015 in Antalya, Akdeniz University. In our study, we aimed to compare anxiety and depression status of severe asthma patients having omalizumab treatment with pretreatment period. For this purpose, the patients were asked to fill in the questionnaire. 

Anxiety level was determined with STAI, whereas depression level was evaluated by BDS. Patients were asked to mark the questionnaires to reflect their emotions they are feeling in the current moment. All patients having omalizumab treatment were included in the study. Patients with known neuropsychiatric disorder were excluded from the study. 

The study has been approved by the Ethics Committee of Akdeniz University. 

**Assessment of the tests:**

Beck Depression Scale: BDS is a scale composed of 21 questions and was developed for measuring emotional, cognitive and somatic motivation components. Also, BDS has been proven for Turkish population in matters of reliability and validity. In the scale, two questions provide emotional areas whereas five somatic signs, 11 cognitive functions, two behaviors and one question evaluate signs among people. 

The survey provides information about itself and widely used it in clinical practice and trials. Patients mark most suitable states for themselves. Each question is scored as 0,1,2 or 3 and total scores are between 0 and 63. In assessment; 0-9 point shows minimal depression 10-18 mild, 19-29 moderate and 30-63 severe (10). 

State Trait Anxiety Inventory: (STAI) is a self-assessing scale developed by Spielberger et al. and is composed of 2 scales of 20 questions (10). First part shows static anxiety and includes questions as ‘How do you feel now?’ Each question has four response options like; any, some, very much and completely. Second part of the test is composed of questions about continuous anxiety (For example; ‘mostly I feel good.’). 

The total score is between 20 and 80. Higher score indicates higher anxiety level. Turkish validation and reliability study was performed by Öner and Le Compte (12). 

**Statistical analysis: **The results are reported as the mean and categorical variables are given as percentages. The normality of distribution was confirmed by the Shapiro-Wilk's W-test. Since Shapiro–Wilk's test results showed fitting to observed data against a normal distribution, statistical analysis of clinical data between two groups consisted of unpaired t-tests, whereas the chi-square/Fisher's exact tests were used for categorical variables. 

Analysis was performed using IBM SPSS statistics for Windows, Version 20.0 (IBM Corp. Released 2013. Armonk, NY: IBM Corp.) software and two-tailed p-value less than 0.05 was considered statistically significant.

## Results

A total of 20 patients with mean age of 50.25 years were included in the study. Gender distribution was 5 (25%) male patients and 15(75%) female patients. All patients had severe asthma diagnosis and had omalizumab treatment. Period of omalizumab use was as mean 17.6 months (2-40 months). Before omalizumab treatment, asthma control level was poor in all patients. 

After omalizumab treatment, all patients’ asthma control levels improved in the level of good control of 70% comobidities, the most types were hypertension and diabetes mellitus, whereas, 14 patients had at least one comorbidity. In anxiety scales, there was statistically significant difference as compared with pretreatment and post treatment periods (table 1). 

**Table 1:** Comparison of anxiety scales of the patients before and after omalizumab treatment

	**Pretreatment**	**Posttreatment**	**p-value**
Beck depression scale	25.35	8.55	P<0.001
Static scale	53.20	37.65	P<0.001
Continuous scale	52.05	42.95	P<0.001

**Figure 1:**
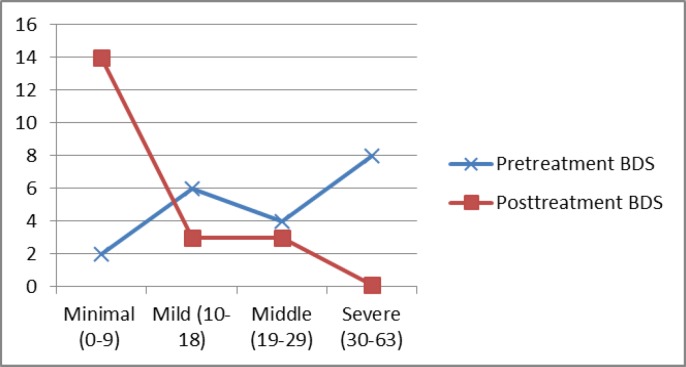
Beck Depression Scale of the patients before and after treatment

Depression (moderate to severe) requires treatment which was present in 12 patients before omalizumab treatment, whereas there were three patients after omalizumab treatment (figure 1). 

Participants' static and continuous anxiety scores decreased significantly after omalizumab treatment compared with the scores before treatment. Except for one patient, every participant (95%) had been using inhalant bronchodilator therapy with montelukast before and after omalizumab treatment.

## Discussion

Asthma is the most common chronic respiratory disease in the world. Good control of asthma is the first line aim of the treatment (13). If asthma is not controlled, it can cause depressive signs and worsens the quality of life. Most severe asthma patients show depressive and anxious signs (14-15). However, it is not clear that these signs worsen asthma severity or asthma causes these signs (16-17). In our study, severe asthma patients had omalizumab treatment in concordance with Global Initiative for Asthma (GINA) guidelines and the study revealed that anxiety and depression levels improved with symptom control and treatment. 

In literature, psychological disorders in asthma patients vary about 11% and 85% (18). In a study with 406 asthma patients; in 34% participants, there was one or more psychiatric disorders. Mostly observed psychiatric disorders in such patients were stated as anxiety disorders. Most seen mood disorder was major depression (14, 16, 19). Katrz et al. performed a cohort study of 439 mostly female adult asthma patients and ofter observed depression, more than two times it compared with normal population. That study stated that worsening of asthma control is associated with depression (20). From Brazil, Vieira et al. reported that the prevalence of psychiatric disorders in uncontrolled asthma patients is significantly higher. In such patients, besides controlling asthma, mood and effect must be examined (14).

Asthma is a chronic disorder and in these patients because of chronic illness social isolation, self-limiting, characteristics and advanced age can cause depressive disorders. Severe asthma and depression can coexist often and both conditions show synergistic effect and can worsen the prognosis (21). Bozbaş et al. observed negative correlation between asthma control level and Beck Depression Scale score in their study. In the same study, it was stated that while treatment assessment of asthma patients; quality of life and psychological status must be evaluated as well (9).

 In our study, all patients had severe asthma diagnosis and it was more often the female gender. Before omalizumab treatment, clinical significant depression was observed in 60% (19) patients. After omalizumab treatment, there was no severe depression, whereas in 3 (1,5%) patients, we observed moderate depression. This observation shows that omalizumab treatment improves depression while controlling asthma.

In literature, it was reported that while severity of chronic illnesses increases, prevalence of psychiatric disorders, anxiety, behavioral disorders also increase. Severe asthma patients claimed as being susceptible to anxiety disorders genetically (22). Anxiety was reported more often in late onset non- eosinophilic asthma phenotype (17). Giacco et al. reported that asthma patients had three times increased anxiety disorder risk during life (16).

 In our study, both static and continuous anxiety scales were significantly higher before omalizumab treatment. Patients stated feeling better and enjoying more life after omalizumab treatment. 

In our study, montelukast as a predisposingt agent for depression was present in about all patients. Despite montelukast usage after controlling asthma depression, anxiety scale scores significantly improved in our study. Most important limitation of our study is being retrospective and having insufficient pupliation. Despite these facts, our study has benefits because of its being the first study evaluating anxiety and depression in asthma patients with omalizumab treatment.

In conclusion, uncontrolled asthma as a chronic disorder can cause depressive symptoms and worsen quality of life. We think that with asthma control the quality of life of such patients can improve. In appropriate indication, omalizumab treatment can improve depression and anxiety in asthma patients.
